# Bis(2,2′-bipyridyl-κ^2^ *N*,*N*′)dichlorido­rhodium(III) perchlorate

**DOI:** 10.1107/S1600536812018685

**Published:** 2012-05-02

**Authors:** Alla Dikhtiarenko, Laura Torre-Fernández, Santiago García-Granda, José R. García, José Gimeno

**Affiliations:** a Departamento Química Orgánica e Inorgánica, Universidad de Oviedo, 33006 Oviedo, Spain; b Departamento de Química Física y Analítica, Facultad de Química, Universidad de Oviedo–CINN, C/ Julián Clavería, 8, 33006 Oviedo, Asturias, Spain

## Abstract

The asymmetric unit of the title compound, [RhCl_2_(C_10_H_8_N_2_)_2_]ClO_4_, consists of one unit of the cationic complex [RhCl_2_(bipy)_2_]^+^ and one uncoordinated perchlorate anion. The Rh^III^ atom is coordinated by four N atoms from two bipyridyl ligands and two Cl atoms, forming a distorted octa­hedral environment. The Cl ligands are *cis*. Two intramolecular C—H⋯Cl hydrogen bonds occur in the cationic complex . In the crystal, mol­ecules are linked together by a hydrogen-bond network involving the H atoms of bipyridyl rings and perchlorate anions. An O atom of the perchlorate anion is disordered over two sites, with an occupancy-factor ratio of 0.78 (3):0.22 (3).

## Related literature

For potential applications of noble metal complexes of pyridyl ligands in biochemistry, catalysis and anti­cancer activity, see: Chifotides *et al.* (2004 ▶); Mbaye *et al.* (2003 ▶); Karidi *et al.* (2005 ▶); Tan *et al.* (2005 ▶). For their photochemical and photophysical properties, see: Forster & Rund (2003 ▶); Arachchige *et al.* (2008 ▶) and for their electrochemical properties, see: Rasmussen *et al.* (1990 ▶). For related structures, see: Al-Noaimi & Haddad (2007 ▶); Andansen & Josephsen (1971 ▶); Choudhury *et al.* (2006 ▶); De Munno *et al.* (1993 ▶); Figgis *et al.* (1985 ▶); Fontaine (2001 ▶); Gao & Ng (2010 ▶); Kramer & Straehle (1986 ▶); Sofetis *et al.* (2006 ▶); Strenger *et al.* (2000 ▶). For similar structures with platinum group metals, see: Lahuerta *et al.* (1991 ▶); Kim *et al.* (2009 ▶); Helberg *et al.* (1996 ▶); Prajapati *et al.* (2008 ▶); Eggleston *et al.* (1985 ▶).
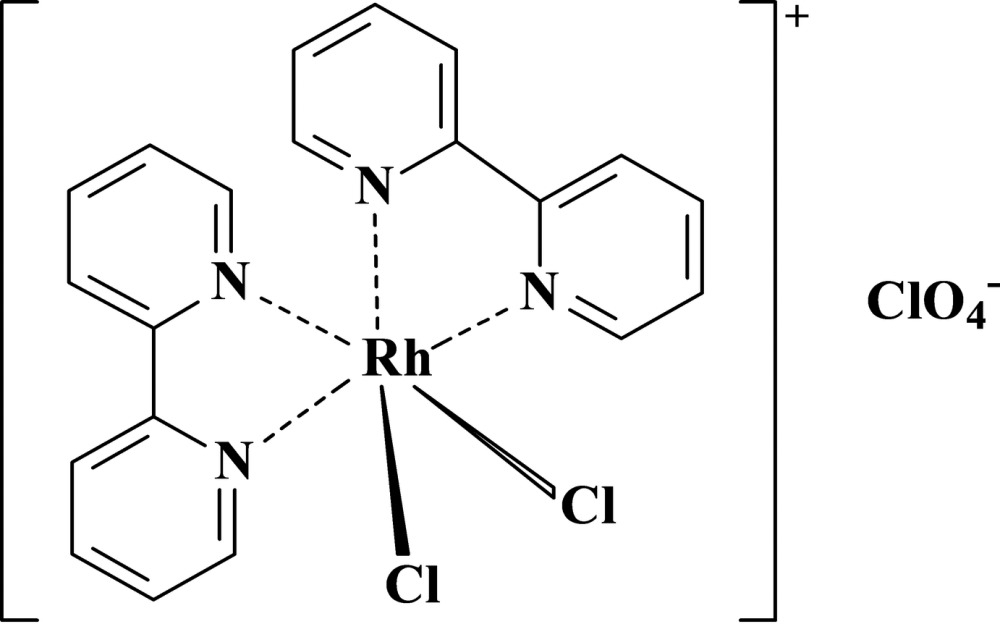


## Experimental

### 

#### Crystal data

[RhCl_2_(C_10_H_8_N_2_)_2_]ClO_4_
*M* *_r_* = 585.63
Orthorhombic, 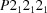

*a* = 11.0344 (2) Å
*b* = 11.6796 (2) Å
*c* = 17.0884 (3) Å
*V* = 2202.33 (8) Å^3^
*Z* = 4
Mo *K*α radiation
μ = 1.18 mm^−1^
*T* = 293 K
0.19 × 0.16 × 0.12 mm

#### Data collection

Agilent Xcalibur Ruby Gemini diffractometer
Absorption correction: multi-scan (*CrysAlis PRO*; Agilent, 2011 ▶) *T*_min_ = 0.973, *T*_max_ = 1
12115 measured reflections
6922 independent reflections
5569 reflections with *I* > 2σ(*I*)
*R*_int_ = 0.026

#### Refinement

*R*[*F* ^2^ > 2σ(*F* ^2^)] = 0.042
*wR*(*F* ^2^) = 0.081
*S* = 1.02
6922 reflections
294 parameters
H-atom parameters constrained
Δρ_max_ = 0.75 e Å^−3^
Δρ_min_ = −0.25 e Å^−3^
Absolute structure: Flack (1983 ▶), 2630 Friedel pairs
Flack parameter: 0.47 (3)

### 

Data collection: *CrysAlis PRO* (Agilent, 2011 ▶); cell refinement: *CrysAlis PRO*; data reduction: *CrysAlis PRO*; program(s) used to solve structure: *SIR92* (Altomare *et al.*, 1994 ▶); program(s) used to refine structure: *SHELXL97* (Sheldrick, 2008 ▶); molecular graphics: *Mercury* (Macrae *et al.*, 2006 ▶); software used to prepare material for publication: *WinGX* (Farrugia, 1999 ▶) and *enCIFer* (Allen *et al.*, 2004 ▶).

## Supplementary Material

Crystal structure: contains datablock(s) global, I. DOI: 10.1107/S1600536812018685/lr2059sup1.cif

Structure factors: contains datablock(s) I. DOI: 10.1107/S1600536812018685/lr2059Isup2.hkl

Additional supplementary materials:  crystallographic information; 3D view; checkCIF report

## Figures and Tables

**Table:** 

Rh1—N2	2.019 (2)
Rh1—N1	2.023 (2)
Rh1—N3	2.037 (2)
Rh1—N4	2.038 (3)
Rh1—Cl3	2.3291 (9)
Rh1—Cl2	2.3344 (9)

**Table:** 

N1—Rh1—N3	174.22 (10)
Cl3—Rh1—Cl2	91.18 (4)

**Table 2:** Hydrogen-bond geometry (Å, °)

*D*—H⋯*A*	*D*—H	H⋯*A*	*D*⋯*A*	*D*—H⋯*A*
C1—H1⋯Cl2	0.93	2.70	3.301 (4)	123
C11—H11⋯Cl3	0.93	2.76	3.358 (4)	123
C3—H3⋯O1^i^	0.93	2.29	3.192 (5)	164
C8—H8⋯O1^ii^	0.93	2.56	3.142 (6)	121
C9—H9⋯O1^ii^	0.93	2.58	3.154 (6)	120
C13—H13⋯O4^iii^	0.93	2.56	3.427 (6)	155

Symmetry codes: (i) 
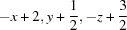
; (ii) 
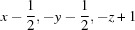
; (iii) 
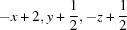
.

**Fig. 1.**
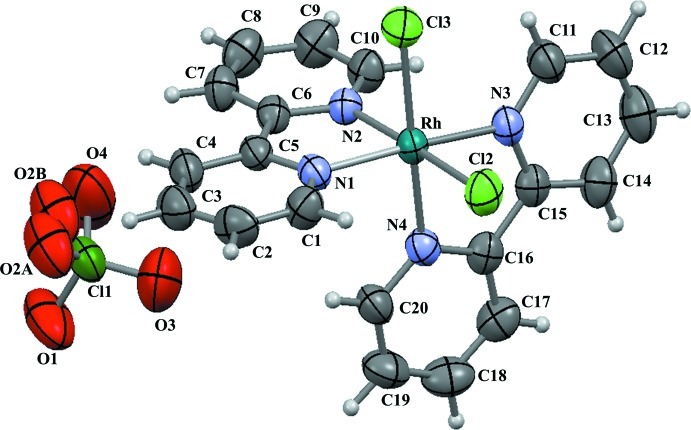
A view of [RhCl2(C10H8N2)2]ClO4 asymmetric unit. Displacement ellipsoids are drawn at the 50% probability level.

## supplementary crystallographic information

### Comment

In recent years, noble metal complexes of pyridyl ligands have received much attention because of their rich electrochemical (Rasmussen *et al.*, 1990, photophysical (Forster & Rund, 2003) and photochemical (Arachchige *et al.*, 2008) properties, and their potential applications in catalysis (Mbaye *et al.*, 2003), biochemistry (Tan *et al.*, 2005; Chifotides *et al.*, 2004) and anticancer activity (Karidi *et al.*, 2005). Bipyridine (bipy) is one of the most commonly used bidentate ligand of this type in the formation of wide variety of transition metal complexes with a general formula of [*M*^II^(bipy)_2_*X*_2_] (*M* = Co, Ni, Mn, Fe) in which *X* is an coordinated anionic ligand such as CN, SCN and chloride (De Munno *et al.*, 1993; Eggleston *et al.*, 1985; Kramer & Straehle, 1986; Al-Noaimi and Haddad, 2007; Fontaine, 2001; Choudhury *et al.*, 2006; Gao & Ng, 2010) and complexes with cationic part [*M*^III^Cl_2_(bipy)_2_]^+^ (*M* = Re, Ru, Co, Ga) and any counter anion like Cl^-^, PF_6_^-^ (Figgis *et al.*, 1985; Sofetis *et al.*, 2006; Andansen & Josephsen, 1971; Strenger *et al.*, 2000; Kim *et al.*, 2009; Prajapati *et al.*, 2008; Helberg *et al.*, 1996). The complex [RhCl_2_(bipy)_2_]Cl.2H_2_O has also been obtained and crystallographycaly determined by Lahuerta *et al.*, 1991. Yet, no crystal structure has been reported for the cationic complex *cis*-[Rh(bipy)_2_Cl _2_]^+^ in its perchlorate form as counter anion, therefore, we report the crystal structure of compound (I).

Complex (I) crystallizes in the orthorhombic space group *P*2_1_2_1_2_1_. The molecular structure of (I) depicted in Figure 1. It has a distorted octahedral geometry with the two chloride ions in *cis* positions. Selected bond lengths for the complex are given in Table 1. The Rh–N axial bond distance (2.038 (3) Å) is slightly longer than Rh–N equatorial bonds (average 2.026 (5) Å). Its may be well compared with the negligible difference between equatorial and axial M–N bonds distances seen in the analogous complexes of platinum metal group (Lahuerta *et al.*, 1991; Kim *et al.*, 2009; Helberg *et al.*, 1996; Prajapati *et al.*, 2008; Eggleston *et al.*, 1985), but greater distortion observed in majority of transition metal complexes (Strenger *et al.*, 2000; Fontaine, 2001; Kramer & Straehle, 1986; Sofetis *et al.*, 2006; Figgis *et al.*, 1985; Choudhury *et al.*, 2006; Gao & Ng, 2010). The Rh–Cl bond distances in (I) are 2.3291 (9) (equatorial) and 2.3344 (9) Å (axial). The N_eq_–Rh–N_eq_ angle is 174.22 (10)° and its distorted from linearity by approximately 6 °. Also Cl_eq_–Rh–Cl_ax_ angle (91.18 (4) °) is nearly octahedral. The *cis* isomerization of cationic complex [RhCl_2_(bipy)_2_]^+^ is stabilized by short contacts. Coordinated chlorides which situated in *cis* position to respect to each other make up short contact Cl2···H1 and Cl3···H11 with distances 2.7 and 2.76 Å, respectively (Tabl. 2). The crystal lattice of (I) is made up of well separated ClO_4_^-^ anions and [RhCl_2_(bipy)_2_]^+^ cations. The perchlorate anion contribute to forming extensive hydrogen bonding net linked together cationic and anionic parts of the structure. The interactions involve the hydrogen bonding between H atoms of bipy ring with oxygen atoms uncoordinated perchlorate anion. Each oxygen atoms participates in four hydrogen bonds to H atoms of aryl groups (Tabl. 2). The atom O2 of the perchlorate anion is disordered and splited over two sites with refined occupancy ratio of 0.78 (3):0.22 (3).

### Experimental

To a solution of RhCl_3_.xH_2_O (0.05 g, 0.231 mmol) and KClO_4_ (0.09 g, 0.693 mmol) in H_2_O (10 ml) was added 2,2'-bipyridine (0.04 g, 0.462 mmol) in CH_3_OH (10 ml), and was stirred for 2 h to yellow solution resulted. Yellowish rhombohedral crystals suitable for X-ray analysis were obtained by slow evaporation during two weeks. RhCl_3_.xH_2_O purchased from Johnson Matthey, all other reagents was obtained commercially from Sigma-Aldrich and used without futher purification.

### Refinement

H atoms were positioned geometrically and allowed to ride on their respective parent atoms [C—H = 0.93 Å and *U*_iso_(H) = 1.2*U*_eq_(C)]. The value of the Flack parameter, 0.47 (3) suggests that the crystal is a racemic twin.

### Crystal data

**Table:**

[RhCl_2_(C_10_H_8_N_2_)_2_]ClO_4_	*F*(000) = 1168
*M**_r_* = 585.63	*D*_x_ = 1.766 Mg m^−^^3^
Orthorhombic, *P*2_1_2_1_2_1_	Mo *K*α radiation, λ = 0.7107 Å
Hall symbol: P 2ac 2ab	Cell parameters from 4926 reflections
*a* = 11.0344 (2) Å	θ = 3.5–32.1°
*b* = 11.6796 (2) Å	µ = 1.18 mm^−^^1^
*c* = 17.0884 (3) Å	*T* = 293 K
*V* = 2202.33 (8) Å^3^	Rhombohedron, yellow
*Z* = 4	0.19 × 0.16 × 0.12 mm

### Data collection

**Table:**

Agilent Xcalibur Ruby Gemini diffractometer	6922 independent reflections
Radiation source: Enhance (Mo) X-ray Source	5569 reflections with *I* > 2σ(*I*)
Graphite monochromator	*R*_int_ = 0.026
Detector resolution: 10.2673 pixels mm^-1^	θ_max_ = 32.1°, θ_min_ = 3.5°
ω scans	*h* = −8→16
Absorption correction: multi-scan (*CrysAlis PRO*; Agilent, 2011)	*k* = −17→10
*T*_min_ = 0.973, *T*_max_ = 1	*l* = −22→25
12115 measured reflections	

### Refinement

**Table:**

Refinement on *F*^2^	H-atom parameters constrained
Least-squares matrix: full	*w* = 1/[σ^2^(*F*_o_^2^) + (0.0292*P*)^2^ + 0.1669*P*] where *P* = (*F*_o_^2^ + 2*F*_c_^2^)/3
*R*[*F*^2^ > 2σ(*F*^2^)] = 0.042	(Δ/σ)_max_ = 0.001
*wR*(*F*^2^) = 0.081	Δρ_max_ = 0.75 e Å^−^^3^
*S* = 1.02	Δρ_min_ = −0.25 e Å^−^^3^
6922 reflections	Absolute structure: Flack (1983), 2630 Friedel pairs
294 parameters	Flack parameter: 0.47 (3)
0 restraints	

### Special details

**Table:**

Geometry. All e.s.d.'s (except the e.s.d. in the dihedral angle between two l.s. planes) are estimated using the full covariance matrix. The cell e.s.d.'s are taken into account individually in the estimation of e.s.d.'s in distances, angles and torsion angles; correlations between e.s.d.'s in cell parameters are only used when they are defined by crystal symmetry. An approximate (isotropic) treatment of cell e.s.d.'s is used for estimating e.s.d.'s involving l.s. planes.
Refinement. Refinement of *F*^2^ against ALL reflections. The weighted *R*-factor *wR* and goodness of fit *S* are based on *F*^2^, conventional *R*-factors *R* are based on *F*, with *F* set to zero for negative *F*^2^. The threshold expression of *F*^2^ > σ(*F*^2^) is used only for calculating *R*-factors(gt) *etc*. and is not relevant to the choice of reflections for refinement. *R*-factors based on *F*^2^ are statistically about twice as large as those based on *F*, and *R*- factors based on ALL data will be even larger.

### Fractional atomic coordinates and isotropic or equivalent isotropic displacement parameters (Å^2^)

**Table:**

	*x*	*y*	*z*	*U*_iso_*/*U*_eq_	Occ. (<1)
Rh1	0.98325 (2)	0.241474 (19)	0.367163 (12)	0.04095 (7)	
Cl1	0.97063 (10)	−0.18258 (8)	0.63515 (5)	0.0629 (2)	
Cl2	1.10302 (9)	0.40548 (7)	0.35644 (6)	0.0609 (2)	
Cl3	0.80775 (9)	0.35183 (8)	0.37395 (6)	0.0603 (2)	
N1	0.9798 (2)	0.2369 (2)	0.48547 (13)	0.0439 (5)	
N2	0.8794 (2)	0.1010 (2)	0.38286 (14)	0.0426 (6)	
N3	0.9942 (3)	0.2300 (2)	0.24844 (13)	0.0467 (6)	
N4	1.1338 (3)	0.1414 (2)	0.35733 (15)	0.0455 (6)	
O1	1.0370 (4)	−0.2722 (3)	0.6687 (2)	0.1228 (15)	
O2A	0.9396 (14)	−0.1068 (7)	0.6981 (7)	0.102 (3)	0.78 (3)
O2B	0.891 (4)	−0.123 (3)	0.6744 (18)	0.102 (3)	0.22 (3)
O3	1.0402 (4)	−0.1234 (3)	0.57914 (19)	0.1075 (12)	
O4	0.8704 (4)	−0.2320 (5)	0.5957 (2)	0.1495 (17)	
C1	1.0374 (3)	0.3097 (3)	0.5332 (2)	0.0557 (9)	
H1	1.0805	0.3705	0.5117	0.067*	
C2	1.0344 (4)	0.2968 (4)	0.6128 (2)	0.0654 (11)	
H2	1.0755	0.3481	0.6448	0.078*	
C3	0.9706 (4)	0.2082 (4)	0.6450 (2)	0.0672 (11)	
H3	0.968	0.1985	0.699	0.081*	
C4	0.9098 (4)	0.1331 (3)	0.59588 (19)	0.0586 (9)	
H4	0.8657	0.0725	0.6166	0.07*	
C5	0.9153 (3)	0.1492 (3)	0.51615 (17)	0.0446 (7)	
C6	0.8570 (3)	0.0741 (2)	0.45826 (17)	0.0442 (7)	
C7	0.7849 (4)	−0.0179 (3)	0.4774 (2)	0.0546 (9)	
H7	0.7708	−0.036	0.5297	0.065*	
C8	0.7341 (4)	−0.0825 (3)	0.4193 (2)	0.0665 (11)	
H8	0.6858	−0.1452	0.4316	0.08*	
C9	0.7550 (4)	−0.0539 (3)	0.3429 (2)	0.0637 (10)	
H9	0.72	−0.0963	0.3028	0.076*	
C10	0.8279 (3)	0.0380 (3)	0.3260 (2)	0.0535 (9)	
H10	0.8421	0.0572	0.274	0.064*	
C11	0.9169 (4)	0.2771 (3)	0.1977 (2)	0.0629 (10)	
H11	0.8496	0.3163	0.2166	0.075*	
C12	0.9344 (4)	0.2689 (4)	0.1183 (2)	0.0719 (11)	
H12	0.8797	0.3022	0.0837	0.086*	
C13	1.0325 (5)	0.2118 (3)	0.0909 (2)	0.0726 (13)	
H13	1.0453	0.2058	0.0373	0.087*	
C14	1.1141 (4)	0.1621 (3)	0.1429 (2)	0.0632 (10)	
H14	1.182	0.1231	0.1249	0.076*	
C15	1.0912 (3)	0.1724 (3)	0.22194 (18)	0.0461 (8)	
C16	1.1686 (3)	0.1207 (3)	0.28234 (18)	0.0464 (8)	
C17	1.2693 (4)	0.0539 (3)	0.2680 (2)	0.0595 (9)	
H17	1.2956	0.0422	0.2169	0.071*	
C18	1.3302 (4)	0.0049 (3)	0.3289 (3)	0.0698 (12)	
H18	1.3968	−0.042	0.3196	0.084*	
C19	1.2925 (4)	0.0256 (3)	0.4035 (3)	0.0686 (11)	
H19	1.3331	−0.0074	0.4455	0.082*	
C20	1.1956 (4)	0.0945 (3)	0.4161 (2)	0.0563 (9)	
H20	1.1715	0.1094	0.4673	0.068*	

### Atomic displacement parameters (Å^2^)

**Table:**

	*U*^11^	*U*^22^	*U*^33^	*U*^12^	*U*^13^	*U*^23^
Rh1	0.04542 (12)	0.04254 (12)	0.03489 (10)	−0.00107 (11)	−0.00011 (10)	0.00272 (10)
Cl1	0.0798 (6)	0.0651 (5)	0.0438 (4)	0.0109 (5)	−0.0048 (5)	0.0050 (4)
Cl2	0.0664 (6)	0.0557 (5)	0.0606 (5)	−0.0147 (4)	0.0015 (5)	0.0072 (4)
Cl3	0.0580 (5)	0.0612 (5)	0.0618 (5)	0.0098 (4)	0.0026 (5)	0.0058 (5)
N1	0.0474 (14)	0.0471 (13)	0.0370 (11)	0.0040 (18)	−0.0028 (10)	0.0026 (10)
N2	0.0443 (15)	0.0422 (13)	0.0414 (15)	−0.0005 (11)	0.0015 (12)	0.0017 (11)
N3	0.0537 (16)	0.0491 (14)	0.0375 (12)	−0.0050 (17)	−0.0008 (11)	0.0049 (11)
N4	0.0483 (16)	0.0430 (13)	0.0451 (15)	−0.0016 (12)	−0.0021 (13)	0.0042 (12)
O1	0.157 (4)	0.120 (3)	0.091 (2)	0.073 (3)	0.009 (2)	0.032 (2)
O2A	0.160 (8)	0.084 (3)	0.063 (4)	0.040 (4)	0.005 (4)	−0.011 (3)
O2B	0.160 (8)	0.084 (3)	0.063 (4)	0.040 (4)	0.005 (4)	−0.011 (3)
O3	0.123 (3)	0.108 (3)	0.092 (2)	−0.013 (2)	0.025 (2)	0.0163 (19)
O4	0.137 (4)	0.207 (5)	0.104 (3)	−0.053 (4)	−0.027 (3)	0.028 (3)
C1	0.057 (2)	0.062 (2)	0.0480 (18)	−0.0090 (18)	0.0027 (17)	−0.0091 (15)
C2	0.063 (3)	0.085 (3)	0.048 (2)	0.002 (2)	−0.0115 (18)	−0.0183 (19)
C3	0.070 (3)	0.090 (3)	0.0414 (18)	0.009 (2)	−0.0019 (19)	−0.0028 (17)
C4	0.069 (3)	0.065 (2)	0.0419 (18)	0.008 (2)	0.0038 (18)	0.0098 (17)
C5	0.0456 (19)	0.0481 (17)	0.0401 (16)	0.0076 (15)	0.0025 (14)	0.0040 (14)
C6	0.051 (2)	0.0390 (16)	0.0432 (16)	0.0056 (15)	0.0066 (15)	0.0031 (13)
C7	0.067 (3)	0.0458 (19)	0.0512 (18)	−0.0012 (17)	0.0086 (18)	0.0064 (16)
C8	0.071 (3)	0.049 (2)	0.079 (3)	−0.0105 (19)	0.011 (2)	0.0006 (19)
C9	0.069 (3)	0.058 (2)	0.063 (2)	−0.009 (2)	0.0035 (19)	−0.0161 (19)
C10	0.060 (2)	0.056 (2)	0.0453 (18)	−0.0061 (18)	0.0009 (17)	−0.0073 (16)
C11	0.071 (3)	0.069 (2)	0.0481 (19)	0.004 (2)	−0.0041 (18)	0.0063 (17)
C12	0.090 (3)	0.079 (3)	0.046 (2)	0.004 (2)	−0.0136 (19)	0.007 (2)
C13	0.108 (4)	0.070 (2)	0.0402 (19)	−0.006 (2)	0.002 (2)	−0.0029 (17)
C14	0.081 (3)	0.062 (2)	0.047 (2)	−0.004 (2)	0.011 (2)	−0.0068 (17)
C15	0.056 (2)	0.0385 (16)	0.0441 (17)	−0.0077 (15)	0.0018 (16)	−0.0020 (13)
C16	0.054 (2)	0.0384 (16)	0.0467 (18)	−0.0089 (15)	0.0023 (15)	−0.0056 (13)
C17	0.060 (2)	0.055 (2)	0.063 (2)	−0.0002 (19)	0.0051 (19)	−0.0151 (18)
C18	0.057 (3)	0.054 (2)	0.098 (3)	0.0120 (19)	−0.003 (2)	−0.013 (2)
C19	0.061 (3)	0.061 (2)	0.084 (3)	0.012 (2)	−0.014 (2)	0.011 (2)
C20	0.060 (2)	0.059 (2)	0.050 (2)	0.0023 (18)	−0.0066 (18)	0.0101 (17)

### Geometric parameters (Å, º)

**Table:**

Rh1—N2	2.019 (2)	C4—H4	0.93
Rh1—N1	2.023 (2)	C5—C6	1.470 (4)
Rh1—N3	2.037 (2)	C6—C7	1.377 (5)
Rh1—N4	2.038 (3)	C7—C8	1.369 (5)
Rh1—Cl3	2.3291 (9)	C7—H7	0.93
Rh1—Cl3	2.3291 (9)	C8—C9	1.366 (5)
Rh1—Cl2	2.3344 (9)	C8—H8	0.93
Rh1—Cl2	2.3344 (9)	C9—C10	1.373 (5)
Cl1—O2B	1.31 (3)	C9—H9	0.93
Cl1—O1	1.401 (3)	C10—H10	0.93
Cl1—O3	1.408 (3)	C11—C12	1.375 (5)
Cl1—O4	1.418 (4)	C11—H11	0.93
Cl1—O2A	1.434 (9)	C12—C13	1.354 (6)
N1—C1	1.338 (4)	C12—H12	0.93
N1—C5	1.353 (4)	C13—C14	1.392 (6)
N2—C10	1.344 (4)	C13—H13	0.93
N2—C6	1.349 (4)	C14—C15	1.379 (5)
N3—C11	1.335 (4)	C14—H14	0.93
N3—C15	1.343 (4)	C15—C16	1.469 (5)
N4—C20	1.332 (4)	C16—C17	1.380 (5)
N4—C16	1.359 (4)	C17—C18	1.365 (5)
C1—C2	1.369 (5)	C17—H17	0.93
C1—H1	0.93	C18—C19	1.362 (6)
C2—C3	1.367 (6)	C18—H18	0.93
C2—H2	0.93	C19—C20	1.356 (5)
C3—C4	1.387 (5)	C19—H19	0.93
C3—H3	0.93	C20—H20	0.93
C4—C5	1.377 (4)		
			
N2—Rh1—N1	80.50 (10)	C2—C3—C4	118.9 (3)
N2—Rh1—N3	96.45 (10)	C2—C3—H3	120.5
N1—Rh1—N3	174.22 (10)	C4—C3—H3	120.5
N2—Rh1—N4	90.41 (11)	C5—C4—C3	119.5 (4)
N1—Rh1—N4	94.74 (10)	C5—C4—H4	120.3
N3—Rh1—N4	80.32 (11)	C3—C4—H4	120.3
N2—Rh1—Cl3	88.36 (8)	N1—C5—C4	120.6 (3)
N1—Rh1—Cl3	87.08 (7)	N1—C5—C6	114.9 (3)
N3—Rh1—Cl3	97.78 (8)	C4—C5—C6	124.4 (3)
N4—Rh1—Cl3	177.61 (8)	N2—C6—C7	121.0 (3)
N2—Rh1—Cl3	88.36 (8)	N2—C6—C5	115.1 (3)
N1—Rh1—Cl3	87.08 (7)	C7—C6—C5	123.9 (3)
N3—Rh1—Cl3	97.78 (8)	C8—C7—C6	119.6 (3)
N4—Rh1—Cl3	177.61 (8)	C8—C7—H7	120.2
Cl3—Rh1—Cl3	0.00 (5)	C6—C7—H7	120.2
N2—Rh1—Cl2	176.85 (7)	C9—C8—C7	119.3 (4)
N1—Rh1—Cl2	96.36 (8)	C9—C8—H8	120.3
N3—Rh1—Cl2	86.69 (7)	C7—C8—H8	120.3
N4—Rh1—Cl2	90.16 (8)	C8—C9—C10	119.4 (4)
Cl3—Rh1—Cl2	91.18 (4)	C8—C9—H9	120.3
Cl3—Rh1—Cl2	91.18 (4)	C10—C9—H9	120.3
N2—Rh1—Cl2	176.85 (7)	N2—C10—C9	121.5 (3)
N1—Rh1—Cl2	96.36 (8)	N2—C10—H10	119.2
N3—Rh1—Cl2	86.69 (7)	C9—C10—H10	119.2
N4—Rh1—Cl2	90.16 (8)	N3—C11—C12	121.5 (4)
Cl3—Rh1—Cl2	91.18 (4)	N3—C11—H11	119.2
Cl3—Rh1—Cl2	91.18 (4)	C12—C11—H11	119.2
Cl2—Rh1—Cl2	0.00 (5)	C13—C12—C11	119.2 (4)
O2B—Cl1—O1	122.6 (14)	C13—C12—H12	120.4
O2B—Cl1—O3	117.0 (14)	C11—C12—H12	120.4
O1—Cl1—O3	111.1 (2)	C12—C13—C14	120.1 (3)
O2B—Cl1—O4	86 (2)	C12—C13—H13	119.9
O1—Cl1—O4	107.4 (3)	C14—C13—H13	119.9
O3—Cl1—O4	107.5 (2)	C15—C14—C13	118.1 (4)
O1—Cl1—O2A	106.2 (5)	C15—C14—H14	121
O3—Cl1—O2A	109.7 (4)	C13—C14—H14	121
O4—Cl1—O2A	115.0 (7)	N3—C15—C14	121.3 (3)
C1—N1—C5	119.7 (3)	N3—C15—C16	115.6 (3)
C1—N1—Rh1	125.7 (2)	C14—C15—C16	123.1 (3)
C5—N1—Rh1	114.7 (2)	N4—C16—C17	119.7 (3)
C10—N2—C6	119.1 (3)	N4—C16—C15	115.2 (3)
C10—N2—Rh1	126.0 (2)	C17—C16—C15	125.1 (3)
C6—N2—Rh1	114.8 (2)	C18—C17—C16	119.9 (4)
C11—N3—C15	119.8 (3)	C18—C17—H17	120.1
C11—N3—Rh1	125.6 (2)	C16—C17—H17	120.1
C15—N3—Rh1	114.6 (2)	C19—C18—C17	119.3 (4)
C20—N4—C16	119.6 (3)	C19—C18—H18	120.4
C20—N4—Rh1	126.2 (2)	C17—C18—H18	120.4
C16—N4—Rh1	114.2 (2)	C20—C19—C18	119.7 (4)
N1—C1—C2	121.6 (3)	C20—C19—H19	120.1
N1—C1—H1	119.2	C18—C19—H19	120.1
C2—C1—H1	119.2	N4—C20—C19	121.8 (4)
C3—C2—C1	119.8 (4)	N4—C20—H20	119.1
C3—C2—H2	120.1	C19—C20—H20	119.1
C1—C2—H2	120.1		
			
N1—Rh1—Cl2—Cl2	0E1 (8)	Cl2—Rh1—N4—C16	−84.4 (2)
N3—Rh1—Cl2—Cl2	0.00 (2)	C5—N1—C1—C2	1.0 (5)
N4—Rh1—Cl2—Cl2	0.00 (2)	Rh1—N1—C1—C2	−176.9 (3)
Cl3—Rh1—Cl2—Cl2	0.00 (2)	N1—C1—C2—C3	−0.4 (6)
Cl3—Rh1—Cl2—Cl2	0.00 (2)	C1—C2—C3—C4	−0.1 (6)
N2—Rh1—Cl3—Cl3	0.00 (3)	C2—C3—C4—C5	0.2 (6)
N1—Rh1—Cl3—Cl3	0.00 (3)	C1—N1—C5—C4	−0.9 (5)
N3—Rh1—Cl3—Cl3	0.00 (3)	Rh1—N1—C5—C4	177.2 (3)
Cl2—Rh1—Cl3—Cl3	0.00 (3)	C1—N1—C5—C6	−179.4 (3)
Cl2—Rh1—Cl3—Cl3	0.00 (3)	Rh1—N1—C5—C6	−1.3 (3)
N2—Rh1—N1—C1	178.2 (3)	C3—C4—C5—N1	0.3 (5)
N4—Rh1—N1—C1	88.5 (3)	C3—C4—C5—C6	178.7 (3)
Cl3—Rh1—N1—C1	−93.0 (3)	C10—N2—C6—C7	1.5 (5)
Cl3—Rh1—N1—C1	−93.0 (3)	Rh1—N2—C6—C7	178.3 (3)
Cl2—Rh1—N1—C1	−2.2 (3)	C10—N2—C6—C5	−178.8 (3)
Cl2—Rh1—N1—C1	−2.2 (3)	Rh1—N2—C6—C5	−2.0 (4)
N2—Rh1—N1—C5	0.2 (2)	N1—C5—C6—N2	2.2 (4)
N4—Rh1—N1—C5	−89.4 (2)	C4—C5—C6—N2	−176.2 (3)
Cl3—Rh1—N1—C5	89.0 (2)	N1—C5—C6—C7	−178.2 (3)
Cl3—Rh1—N1—C5	89.0 (2)	C4—C5—C6—C7	3.4 (5)
Cl2—Rh1—N1—C5	179.9 (2)	N2—C6—C7—C8	−0.7 (6)
Cl2—Rh1—N1—C5	179.9 (2)	C5—C6—C7—C8	179.7 (3)
N1—Rh1—N2—C10	177.6 (3)	C6—C7—C8—C9	−0.6 (6)
N3—Rh1—N2—C10	−7.4 (3)	C7—C8—C9—C10	1.0 (6)
N4—Rh1—N2—C10	−87.7 (3)	C6—N2—C10—C9	−1.1 (5)
Cl3—Rh1—N2—C10	90.3 (3)	Rh1—N2—C10—C9	−177.5 (3)
Cl3—Rh1—N2—C10	90.3 (3)	C8—C9—C10—N2	−0.1 (6)
N1—Rh1—N2—C6	1.1 (2)	C15—N3—C11—C12	−0.5 (5)
N3—Rh1—N2—C6	176.1 (2)	Rh1—N3—C11—C12	177.1 (3)
N4—Rh1—N2—C6	95.8 (2)	N3—C11—C12—C13	0.0 (6)
Cl3—Rh1—N2—C6	−86.3 (2)	C11—C12—C13—C14	0.0 (6)
Cl3—Rh1—N2—C6	−86.3 (2)	C12—C13—C14—C15	0.5 (6)
N2—Rh1—N3—C11	89.5 (3)	C11—N3—C15—C14	1.0 (5)
N4—Rh1—N3—C11	178.8 (3)	Rh1—N3—C15—C14	−176.9 (3)
Cl3—Rh1—N3—C11	0.3 (3)	C11—N3—C15—C16	−178.1 (3)
Cl3—Rh1—N3—C11	0.3 (3)	Rh1—N3—C15—C16	4.0 (3)
Cl2—Rh1—N3—C11	−90.4 (3)	C13—C14—C15—N3	−1.0 (5)
Cl2—Rh1—N3—C11	−90.4 (3)	C13—C14—C15—C16	178.1 (3)
N2—Rh1—N3—C15	−92.7 (2)	C20—N4—C16—C17	−1.7 (5)
N4—Rh1—N3—C15	−3.4 (2)	Rh1—N4—C16—C17	−179.9 (2)
Cl3—Rh1—N3—C15	178.0 (2)	C20—N4—C16—C15	177.4 (3)
Cl3—Rh1—N3—C15	178.0 (2)	Rh1—N4—C16—C15	−0.8 (3)
Cl2—Rh1—N3—C15	87.3 (2)	N3—C15—C16—N4	−2.1 (4)
Cl2—Rh1—N3—C15	87.3 (2)	C14—C15—C16—N4	178.8 (3)
N2—Rh1—N4—C20	−79.4 (3)	N3—C15—C16—C17	177.0 (3)
N1—Rh1—N4—C20	1.1 (3)	C14—C15—C16—C17	−2.2 (5)
N3—Rh1—N4—C20	−175.9 (3)	N4—C16—C17—C18	2.8 (5)
Cl2—Rh1—N4—C20	97.5 (3)	C15—C16—C17—C18	−176.2 (3)
Cl2—Rh1—N4—C20	97.5 (3)	C16—C17—C18—C19	−1.8 (6)
N2—Rh1—N4—C16	98.7 (2)	C17—C18—C19—C20	−0.2 (7)
N1—Rh1—N4—C16	179.2 (2)	C16—N4—C20—C19	−0.4 (5)
N3—Rh1—N4—C16	2.3 (2)	Rh1—N4—C20—C19	177.6 (3)
Cl2—Rh1—N4—C16	−84.4 (2)	C18—C19—C20—N4	1.4 (6)

### Hydrogen-bond geometry (Å, º)

**Table:**

*D*—H···*A*	*D*—H	H···*A*	*D*···*A*	*D*—H···*A*
C1—H1···Cl2	0.93	2.7	3.301 (4)	123
C11—H11···Cl3	0.93	2.76	3.358 (4)	123
C3—H3···O1^i^	0.93	2.29	3.192 (5)	164
C8—H8···O1^ii^	0.93	2.56	3.142 (6)	121
C9—H9···O1^ii^	0.93	2.58	3.154 (6)	120
C13—H13···O4^iii^	0.93	2.56	3.427 (6)	155

Symmetry codes: (i) −*x*+2, *y*+1/2, −*z*+3/2; (ii) *x*−1/2, −*y*−1/2, −*z*+1; (iii) −*x*+2, *y*+1/2, −*z*+1/2.

## References

[bb1] Agilent (2011). *CrysAlis PRO* Agilent Technologies, Yarnton, England.

[bb2] Allen, F. H., Johnson, O., Shields, G. P., Smith, B. R. & Towler, M. (2004). *J. Appl. Cryst.* **37**, 335–338.

[bb3] Al-Noaimi, M. & Haddad, S. F. (2007). *Acta Cryst.* E**63**, m2332.

[bb4] Altomare, A., Cascarano, G., Giacovazzo, C., Guagliardi, A., Burla, M. C., Polidori, G. & Camalli, M. (1994). *J. Appl. Cryst.* **27**, 435.

[bb5] Andansen, P. & Josephsen, J. (1971). *Acta Chem. Scand.* **25**, 3255–3260.

[bb6] Arachchige, S. M., Brown, J. & Brewe, K. J. (2008). *J. Photochem. Photobiol. A*, **197**, 13–17.

[bb7] Chifotides, H. T., Fu, P. K. L., Dunbar, K. R. & Turro, C. (2004). *Inorg. Chem.* **43**, 1175–1183. 10.1021/ic034438m 14753842

[bb8] Choudhury, S. R., Dutta, A., Mukhopadhyay, S., Lu, L.-P. & Zhu, M.-L. (2006). *Acta Cryst.* E**62**, m1489–m1491.

[bb9] De Munno, G., Nicolò, F. & Julve, M. (1993). *Acta Cryst.* C**49**, 1049–1052.

[bb10] Eggleston, D. S., Goldsby, K. A., Hodgson, D. J. & Meyer, T. J. (1985). *Inorg. Chem.* **24**, 4573–4580.

[bb11] Farrugia, L. J. (1999). *J. Appl. Cryst.* **32**, 837–838.

[bb12] Figgis, B. N., Reynolds, P. A. & White, A. H. (1985). *Inorg. Chem.* **24**, 3762–3770.

[bb13] Flack, H. D. (1983). *Acta Cryst.* A**39**, 876–881.

[bb14] Fontaine, F. G. (2001). *Acta Cryst.* E**57**, m270–m271.

[bb15] Forster, L. C. & Rund, J. V. (2003). *Inorg. Chem. Commun.* **6**, 78–81.

[bb16] Gao, S. & Ng, S. W. (2010). *Acta Cryst.* E**66**, m1692. 10.1107/S1600536810049251 PMC3011694 21589347

[bb17] Helberg, L. E., Orth, S. D., Sabat, M. & Harman, W. D. (1996). *Inorg. Chem.* **35**, 5584–5594. 10.1021/ic960001l 11666750

[bb18] Karidi, K., Garoufis, A., Tsipis, A., Hadjiliadis, N., Dulk, H. & Reedijk, J. (2005). *Dalton Trans.* pp. 1176–1187. 10.1039/b418838a 15782252

[bb19] Kim, N.-H., Hwang, I.-C. & Ha, K. (2009). *Acta Cryst.* E**65**, m180. 10.1107/S1600536809000725 PMC2968269 21581784

[bb20] Kramer, T. & Straehle, J. (1986). *Z. Naturforsch. Teil B*, **41**, 692–696.

[bb21] Lahuerta, P., Latorre, J., Martínez-Máñez, R., García-Granda, S. & Gómez-Beltrán, F. (1991). *Acta Cryst.* C**47**, 519–522.

[bb22] Macrae, C. F., Edgington, P. R., McCabe, P., Pidcock, E., Shields, G. P., Taylor, R., Towler, M. & van de Streek, J. (2006). *J. Appl. Cryst.* **39**, 453–457.

[bb23] Mbaye, M. D., Demersement, B., Reneaud, J.-L., Toupet, L. & Bruneau, C. (2003). *Angew. Chem. Int. Ed.* **42**, 5066–5068. 10.1002/anie.200352257 14595634

[bb24] Prajapati, R., Yadan, V. K., Dubey, S. K., Durham, B. & Mishra, L. (2008). *Indian J. Chem. Sect. A*, **47**, 1780–1787.

[bb25] Rasmussen, S. C., Richter, M. M., Yi, E., Place, H. & Brewer, K. J. (1990). *Inorg. Chem.* **29**, 3926–3932.

[bb26] Sheldrick, G. M. (2008). *Acta Cryst.* A**64**, 112–122. 10.1107/S0108767307043930 18156677

[bb27] Sofetis, A., Raptopoulou, C. P., Terzis, A. & Zafiropoulos, T. F. (2006). *Inorg. Chim. Acta*, **359**, 3389–3395.

[bb28] Strenger, I., Rosu, T. & Negoiu, M. (2000). *Z. Kristallogr. New Cryst. Struct.* **215**, 489–490.

[bb29] Tan, L. F., Chao, H., Li, H., Liu, Y. J., Sun, B., Wei, W. & Ji, L. N. (2005). *J. Inorg. Biochem.* **99**, 513–520. 10.1016/j.jinorgbio.2004.10.028 15621284

